# Psychological Variables Associated with HPV Vaccination Intent in Romanian Academic Settings

**DOI:** 10.3390/ijerph18178938

**Published:** 2021-08-25

**Authors:** Liliana Veronica Diaconescu, Iuliana Raluca Gheorghe, Tamara Cheşcheş, Ovidiu Popa-Velea

**Affiliations:** 1 Department of Medical Psychology, Faculty of Medicine, University of Medicine and Pharmacy “Carol Davila”, 050474 Bucharest, Romania; ovidiu.popa-velea@umfcd.ro; 2 Department of Marketing and Medical Technology, Faculty of Medicine, University of Medicine and Pharmacy “Carol Davila”, 050474 Bucharest, Romania; raluca.gheorghe@umfcd.ro; 3 “Sf. Ioan” Emergency Clinical Hospital, 042122 Bucharest, Romania; tamara.chesches@rez.umfcd.ro

**Keywords:** vaccination intent, HPV, coping, sense of coherence, health locus of control, academic

## Abstract

The aim of this study was to evaluate (1) the female medical students’ knowledge about HPV infection; (2) the associations between the HPV vaccination intent and coping strategies, health locus of control (HLOC), and sense of coherence; and (3) the specific differences between preclinical and clinical students in terms of the vaccination intent. Participants included 1243 female medicine students (mean age = 21.526, SD = 2.007), who completed The Multidimensional Health Locus of Control (MHLC)—Form A, the Brief COPE Scale, the Sense of Coherence Scale (SOC-13), and two questionnaires measuring the knowledge about the HPV infection and the HPV vaccination intent. Results show a good knowledge about HPV, which progressively increased during the study cycles. Still, the main contributors to vaccination intent are represented by coping strategies and health locus of control. Refusal of vaccination is associated to behavioral disengagement and the use of religion, precontemplation and contemplation to denial, and preparation to planning, positive reframing, and the powerful others component of HLOC. Sense of coherence did not predict vaccination intent. In clinical years, active coping outweighs HLOC in making the decision to get vaccinated. These results could be helpful in designing personalized strategies for addressing vaccine hesitancy in academic communities.

## 1. Introduction

Human papillomavirus (HPV) is the most commonly sexually transmitted infection (STI) in the world [1] and has the highest prevalence among females aged 20 to 24 years [2]. In terms of mortality, HPV represents the most frequent cause of cervical cancer, especially in less developed countries [3,4,5].

The administration of the HPV vaccine has been reported to decrease the incidence of HPV infections and cervical cancer in general population [6,7,8]. Although the decision to get vaccinated can be dependent on the parental consent in adolescence [9,10], it becomes a matter of self-decision in adult women [11]. Despite this, only 12% of females and 3% of males reported initiating the HPV vaccine in adulthood [12]. A series of factors are worldwide known to account for this phenomenon, such as the lack of awareness about HPV infection [13], the fear of needles and of side effects [11,14,15], mistrust in the HPV vaccine [16,17,18], perceived low risk of contracting HPV and its perceived low severity [15], decreased health care utilization by young people [19], economic disadvantage and low financial income [11], and negative social influence from peers and social media [18,20,21,22,23,24].

In Romania, the age-standardized incidence of cervical cancer is 20 new cases/100,000 women [25], with the highest cervical cancer mortality (14.2 deaths/100,000 women) among the EU countries [26]. Although many Romanian women are aware of the existence of this disease and of the testing process, their knowledge is generally superficial (e.g., 94.5% heard of Pap smear, but only 58.7% know what it actually is) [27]. This attitude is mirrored with the HPV vaccine. A first initiative of HPV vaccination was launched in Romania in 2008, when a free-of-charge vaccination program, coordinated by the Ministry of Health, was organized for all girls aged 10–11. The success rate of the program was, however, very low, with only 2.57% of the eligible girls getting immunized [28]. In 2009, before the campaign was resumed, the Ministry of Health included a preliminary phase of communication about HPV-associated risks and HPV vaccination, but the vaccine coverage remained low (9%) [29]. In January 2020, after an 11-year break, the program was continued, preceded by an information and public awareness campaign about the dangers related to the HPV and the protection offered by the vaccine. This time, the program included an additional support section for physicians and pharmacists in order to help them provide clear and accurate information about HPV vaccination to interested individuals. The vaccination itself was offered to girls aged 11–14 years old whose parents previously requested vaccination [30,31]. The evaluation of this new initiative is still under way, but preliminary assessment indicates a lower rate of vaccination than expected and the need for more active information and preventive measures to accelerate its acceptance. Many subjective reasons could have led to vaccination reluctance, including the overall risky perception of the vaccine, the fear of adverse effects, the novelty of its implementation, the beliefs of associated infertility risks, and the perception of the vaccination procedure as an experiment [32,33]. Globally, in the Romanian population, decisions about HPV vaccination seem mostly influenced by negative emotions (possibly generated by the insufficient information provided by the health system and by a negative campaign carried out by mass media [32,34]), which far outweigh the gaps in knowledge about HPV infection and HPV vaccine [35]. Vulnerable women are exposed to an even higher risk of contracting HPV and may feel embarrassed to freely disclose their frail medical and psychological status [36,37].

While exploring the psychological factors associated with vaccination opposition and vaccine hesitancy, a series of variables appear to be important:

- Health locus of control (HLOC): low levels of internal HLOC may lead to decreased compliance to vaccinations [38]. In contrast, higher values are associated not only to pro-vaccination attitudes but also to preventive behaviors and higher adherence [39];
- Coping strategies: avoidance-focused coping is reported in literature to have a negative impact on the vaccination intent, while more active, problem-focused strategies are related to preventive behavior and a higher propensity to seek vaccination [40,41];
- Sense of coherence (SOC): has a moderating and a mediating effect on health [42]. People with a low SOC tend to be in poorer health [43,44] and rarely display preventive behaviors, such as vaccination; and
- Stages of change: people in the precontemplation stage have no intention to change their behavior [45] and thereby are vaccine hesitant. However, individuals in this group as well as those in the contemplation phase could be subjects of targeted interventions to increase vaccine acceptance.

Beside the aforementioned variables, a considerable barrier in vaccination campaigns is represented by the lack of awareness and knowledge about HPV infection and vaccination [35,46]. As revealed in various studies, in many cases, the knowledge is assessed as poor or inaccurate [47,48]. Inversely, high levels of knowledge along with perceived severity are associated with the increased inclination to get vaccinated [49,50].

In the academic communities, in particularly in medical universities, not many female students talk freely about the health risks associated with their sexual behavior or about their HPV vaccine hesitancy [51] even in self-help therapeutic groups [52,53]. Nevertheless, the investigation into vaccine perception in this population is important for at least three reasons: (a) their averagely higher levels of education allow for a much more sensitive evaluation of the role of psychological variables in their pro- or anti-vaccination intent; (b) these students could represent credible sources of medical information, thereby influencing the choice of other individuals around them to get vaccinated; and (c) they pertain to an age group where the catch-up vaccination is feasible.

The aim of this study was (1) to evaluate the female medical students’ knowledge about HPV infection; (2) to assess the specific differences and the comparative predictive value of coping strategies, health locus of control (HLOC), and sense of coherence in regards to the HPV vaccination intent; and (3) to evaluate the specific differences between vaccination intent in students having an exclusive theoretical knowledge of HPV and those students with clinical experience with HPV infection and consequences, i.e., cervical cancer.

## 2. Materials and Methods

### 2.1. Design

The design of the study was cross-sectional, with a single administration of a series of standardized psychometric instruments.

### 2.2. Participants

In order to ensure representativity of the study sample, we included in the study 1243 medical students (mean age = 21.526, SD = 2.007) from 9 medical universities in three different regions of Romania and Bucharest. We recruited a convenience sample of medical students through posted announcements on their institutional online groups. This process was based on the participants’ availability until the time limit was reached. All participants met the inclusion criteria, set as (1) being female, at least 19 years of age, and having the status of current undergraduate students in the abovementioned institutions and (2) having offered the informed consent to participate in the study. Exclusion criteria were represented by (1) students already vaccinated with one or more doses of the HPV vaccine, (2) students displaying current self-reported somatic or psychiatric morbidity, (3) students with cognitive deficits or any other impairments that would render the understanding and completion of the study questionnaires difficult, and (4) lack of completion of one or more study instruments.

### 2.3. Procedure

Data were collected in May–November 2020 through the administering of an online set of questions containing the study instruments. Before taking part in this research, students who expressed interest in participating received an explanatory statement about the study and completed informed consent forms. Subsequently, they were sent the web link for answering the psychometric instruments and the survey. Procedures in the study were designed in accordance with the World Medical Association Declaration of Helsinki and with the ethical guidelines published by the Committee of Ethics at the University of Medicine and Pharmacy Carol Davila—Bucharest (ethical approval no. 10098/13 May 2020). A researcher (TC) was available by phone or email in case the participants had questions concerning the filling of the questionnaires. All responses were processed anonymously, and a numerical code was assigned for each participant. The collected data were accessible exclusively to study researchers (OPV, IRG, TC, LVD), while regular didactic staff had no access to the nominal distribution, collection, or interpretation of questionnaires. The interpretation of the questionnaires was performed independently by two researchers (IRG, TC) and cross-checked for congruence afterwards. Final results were included in a SPSS 21 (SPSS^®^ Inc., Chicago, IL, USA) database.

### 2.4. Instruments

1. The Multidimensional Health Locus of Control (MHLC)—Form A [54] comprises 18 items in three subscales (“internality”, “powerful others externality”, and “chance externality”), allowing the identification of the predominant source of identified control over one’s health. The answers are given on a 6-point Likert scale (ranging from 1 = “strongly disagree” to 6 = “strongly agree”). The MHLC is used to predict health behaviors, including preventive attitudes, and was reported to display a good reliability (0.69–0.72) [55,56].
2. Brief COPE Scale contains 28 items, which measure 14 distinct coping strategies [57]. The answers are graded on a 4-point Likert scale (from 1 = “I haven’t been doing this at all” to 4 = “I have been doing this a lot”). The test has generally been reported to have good reliability (0.50–0.90) [57,58] and is validated in the Romanian population.
3. The Sense of Coherence Scale—the shorter version with 13 questions (SOC-13) [59] measures how a person handles stress, by referring to the Antonovsky’s definition of SOC [60], consisting of three components: comprehensibility, manageability, and meaningfulness. Comprehensibility deals with the extent to which a person sees the world as ordered and is able to mobilize the resources needed to cope. Manageability refers to understanding the problem and having the necessary resources to cope successfully. Meaningfulness pertains to the belief that coping makes sense and that one wishes to cope. The responses on the scale are placed on a semantic scale of 1 point to 7 points, where 1 and 7 indicate extreme feelings about questions (and statements) about how one’s life is experienced. The total score ranges between 13 and 91 points. The scale has a good Cronbach alpha coefficient of 0.74–0.91 [61].
4. The questionnaire of knowledge about HPV infection, extracted from previous published research on this theme [61], contains 16 items, to which the answers are binary (“true” or “false”). The instrument has a very good internal consistency (Cronbach alpha = 0.849) [62].
5. The questionnaire about the HPV vaccination intent, adapted from an instrument used previously [63] and constructed according to the description of stages in the Transtheoretical Model of Change (TTM) [64], includes 10 items, relevant for:
  - The refusal of vaccination (e.g., “I do not plan to get vaccinated ever against HPV”);
  - Precontemplation (e.g., “Although vaccination is a good thing, I am unsure about my intention to get vaccinated”);
  - Contemplation (e.g., “I am willing to get vaccinated, but I do not plan this in the next 6 months”);
  - Preparation (e.g., “I plan to get vaccinated (first shot) in the next 6 months, but I have not tried yet to schedule an appointment”);
  - Action (e.g., “I have already scheduled an appointment, and I have received at least one shot of the HPV vaccine”).

The questionnaire was reported to display a good content validity [65].

### 2.5. Data Analysis

Descriptive analyses were realized for socio-demographic and psychological variables.

Statistical analysis was performed with SPSS Statistics 21 (SPSS^®^ Inc., Chicago, IL, USA). It comprised one-way ANOVA (followed by Tukey’s post-hoc tests) to compare the differences among study groups and multiple logistic regression to identify the predictors of the vaccination intent for each distinct phase of the TTM model. Additionally, separate *t*-tests for independent samples to compare the results of students in the sixth study year (who have been exposed to both theory and practice regarding HPV and its consequences) to those in the second study year (who have an exclusively theoretical understanding of HPV).

Throughout statistical analyses, missing data were handled through listwise deletion. For all calculations, the threshold of statistical significance was *p* < 0.05.

## 3. Results

### 3.1. Descriptive Data

The study sample was subdivided into three distinct categories according to their responses at the questionnaire about the HPV intent (Table 1):

- Group 1: no vaccination intent: *n* = 196 (15.8%);
- Group 2: the vaccination intent is only theoretical and consists in thoughts about the utility of the vaccine (precontemplation: *n* = 175 (*n* = 14.1%)) or about the personal benefit from getting vaccinated (contemplation: *n* = 546 (43.9%)); and
- Group 3: the vaccination intent is not only theoretical, but it has also a practical component (preparation: *n* = 326 (26.2%)).

The vast majority of the respondents (79.001%) originated in urban areas. A majority of them studied in the Northwest regions of the country (54.625%). Most of the participants were enrolled in the clinical study years (3–6) (63.878%).

Table 2 synthesizes the results obtained by the three study groups at the tests assessing HPV knowledge and psychological variables (coping strategies, health locus of control, and sense of coherence).

Across the whole sample, the most preferred coping strategies were planning, active coping, and acceptance, while the least preferred were substance use and behavioral disengagement. Several differences emerged between groups, with a more substantial use of behavioral disengagement and religion in group 1, denial and seek for instrumental support in group 2, and planning and positive reframing in group 3.

The HPV knowledge was satisfactory in all study groups and the whole sample (range 12.214–12.269 out of 16).

The sense of coherence had a high dispersion of results (range 23–83), with the sample mean (49.134) placed among the low scores reported in literature for academic populations [66,67,68].

In terms of health locus of control, most students placed its main source internally, followed by the powerful others and by chance. This hierarchy was maintained irrespective of the participants’ vaccination intent.

### 3.2. Statistical Differences between Groups

Table 3 displays the significant differences between the three study groups (ANOVA) and the sources of these differences (Tukey’s post-hoc tests).

Students refusing vaccination (group 1) had significantly higher scores in behavioral disengagement and lower scores in positive reinterpretation compared to students preparing for getting vaccinated (group 3). Moreover, they had a significantly higher use of religion compared to students only thinking about vaccination (group 2).

Students in group 3 had significantly lower scores of denial and marginally higher scores of acceptance compared to those in group 2.

All the three groups differed significantly in terms of the role played by the powerful others, this getting progressively more important from group 1 to group 3.

### 3.3. Predictors of the Vaccination Intent

Multinominal regression analysis was used to identify the predictors of the vaccination intent. Results are displayed in Table 4.

The goodness-of-fit of the model was satisfactory (chi-square = 164.710, df = 158, *p* < 0.341).

Across the whole TTM stages, the most important predictors were denial (*p* < 0.003), positive reframing (*p* < 0.010), religion (*p* < 0.013), and health locus of control (the powerful others component) (*p* < 0.001).

Participants in group 1 were characterized, compared to group 3, by higher scores in behavioral disengagement (B = 0.234, standard error = 0.089, df = 1, *p* < 0.008) and religion (B = 0.129, standard error = 0.050, df = 1, *p* < 0.010) and lower scores in positive reframing (B = −0.258, standard error = 0.078, df = 1, *p* < 0.001). In addition, those in group 1 counted less on powerful others (B = −0.112, standard error = 0.020, df = 1, *p* < 0.001).

Similarly, participants in group 2 were characterized, compared to group 3, by lower scores on the powerful others component of the HLOC scale (B = −0.050, standard error = 0.020, df = 1, *p* < 0.014).

### 3.4. Preclinical—Clinical Differences

These differences were evaluated by comparing students in the preclinical cycle (in the second study year, having an exclusively theoretical knowledge of HPV) and students in the clinical cycle (sixth year, with clinical experience with HPV infection and its consequences). The significant results of this analysis are displayed in Table 5.

As expected, students in the sixth study year, irrespective of their vaccination intent, had statistically more HPV knowledge than those in the second year.

Still, the students in the preparation phase had two additional particularities: they tended to follow, in terms of health, an authoritative figure (if they were in the preclinical cycle) or were significantly more active (if they were in the clinical cycle).

## 4. Discussion

This study focused on assessing the female medical students’ knowledge about HPV infection; the specific differences and the comparative predictive value of coping strategies, health locus of control (HLOC), and sense of coherence in regards to the HPV vaccination intent; and the specific differences between vaccination intent in students in the preclinical and clinical study cycles after having been exposed to HPV and its consequences.

The descriptive data illustrated a satisfactory overall level of knowledge about HPV (12.25 out of 16). This finding was to a certain point predictable given that medical students are in direct and frequent contact with health information about HPV and associated risk factors.

The most used coping strategies were generally the adaptive ones (planning, active coping, acceptance, and positive reframing), while the least used were the avoidant ones (denial, behavioral disengagement, substance use). Generally, the use of this kind of strategies is associated, in medical students, with better health outcomes [69]. In the particular case of our study, this may reflect the intention of the participants as a whole to be actively involved in aspects related to their health, including preventive behaviors. From a prognostic perspective, this could be associated in the long term with a lower risk of cervical cancer and/or with a better management of this disease.

Still, significant differences were met between the three study groups in terms of coping strategies preferences. Participants who did not express the intention to get the HPV vaccine (group 1) were characterized by behavioral disengagement and the use of religion, reflecting a higher orientation towards emotion-focused coping in handling difficult, uncertain, or risky circumstances. In contrast, the respondents in the precontemplation and contemplation stages (group 2) used more often denial. In terms of long-term evolution, this could be potentially dangerous, as they may lack awareness about the risk to get the HPV infection and/or cervical cancer and may stagnate in these stages for a long time or even return to the previous one (no vaccination intent). Still, the regular use of instrumental support by these participants could offer reasons of hope, as this could be associated with a higher tendency to ask for help and support if needed. Individuals belonging to the preparation stage (group 3) preferred planning and positive reframing as tools to better handle stressful emotions (such as those related to the risk of HPV infection or to fear of the vaccine’s side effects). These could have helped them to see the HPV-associated risks in more positive and resolvable terms.

In terms of the sense of coherence, this was assessed as lower than normal in all three study groups. Furthermore, the sense of coherence did not prove in our study to be predictive for the vaccination intent; in other words, understanding the link between vaccination and the lower risk of cervical cancer could have not played a significant role in adopting this preventive behavior. These findings come in contrast with other literature studies that illustrate the importance of the sense of coherence for developing the intention to get vaccinated [70]. This contradiction could stem from the large dispersion of the SOC results (possibly reflecting a lack of proper understanding of the test questions) but could be equally explained by the already vaccinated individuals (possibly with a higher SOC) not having taken part in the study. Another possibility for a lower SOC could stem from its general tendency to decrease during the SARS-CoV-2 pandemic, especially in women, on the general background of higher insecurity and contradictory information surrounding COVID-19 [71,72].

Regarding the health locus of control, most of the students had a high level of internal HLC, followed by the powerful others HLC and chance HLC. This evidence is encouraging, as the predominance of the internal HLC could reflect the will and the ability to perform preventive behaviors, such as vaccination. Literature data using standardized instruments also confirm that high internal HLC correlates positively with preventive behaviors [73,74,75,76] and in particular with adherence to the cervical cancer screening programs [77], getting even stronger after vaccination [78]. Oppositely, fatalistic beliefs and/or lack of control are associated with a low adherence to HPV vaccination [79].

In what concerned the contribution brought by the powerful others HLC, this could have played in the studied group an additional role in the decision to actually perform HPV vaccination. This effect, in relation to the specific authority brought by the physicians’ expertise, has been also reported in literature [80]. However, it should be emphasized that this effect is not guaranteed in the long run, as the position of the influential sources regarding vaccination could vary in time or according to circumstances. Similarly, the chance HLC can also represent a factor potentially leading to vaccine hesitancy or denial.

In terms of the predictors of the vaccination intent, the students in group 1 differed from those in group 3 essentially through their higher scores in behavioral disengagement and religion. They seem also to be characterized by a certain psychological rigidity (as they display a low ability of positive reframing) and higher autonomy (as they lean less on powerful others in taking decisions about their health). In contrast, groups 2 and 3 differed essentially only in what concerned the higher importance of powerful others in the decision of getting vaccinated, met by the students from group 3. From this point of view, the personal example of the persons invested with authority (be this formal or informal) seems to be essential for making the transition from precontemplation/contemplation to preparation.

Although the level of knowledge about HPV was satisfactory, it did not represent, as such, a predictor of the vaccination intent. The high level of knowledge about HPV infection seems to be a necessary but not sufficient condition for determining the young, academic population to adopt a preventive HPV vaccine behavior. This result does not replicate the findings of previous studies on the same theme, which illustrate the positive predictive value of the level of HPV knowledge on vaccination intent [81,82].

The specific comparisons realized between students in the preclinical cycle (second study year) and those in the clinical cycle (sixth study year) revealed that medical knowledge does not predict alone the active preparation for vaccination. Important additional elements are represented by the role of the powerful others (in the case of preclinical students) and active coping (in the case of clinical students). These characteristics could be very informative for the key players in the field of education in designing effective personalized programs in favor of vaccination.

### Limits of Research

Our study has several limitations. Despite including students from all study years, it realized only a cross-evaluation of their vaccination intent. The study included only psychological variables and not social ones. Some variables, such as the powerful others HLOC, may have had an ambivalent influence in time on the vaccination intent. To further investigate the predictors of HPV vaccination, future research could include additional psychosocial factors relevant for personality or social conformity as well as social parameters reported in literature to influence the vaccination intent (e.g., income, access to vaccines, or the pervasive nature of restrictive group norms [83,84,85]).

## 5. Conclusions

This study offers evidence about the HPV knowledge and the factors influencing HPV vaccination intent among female medical students. Among these factors, the most important are HLOC (the powerful others component), coping strategies, and the position in the academic cycle.

Consequently, a series of practical aspects emerge as important for ensuring a higher chance of success of HPV vaccination programs in academic settings. This kind of programs should not rely only on information (especially as this is not by itself a predictor of the vaccination intent) but also on psychological variables (such as coping style, health locus of control, and the impact of significant others). They should be doable and flexible and always consider the placement of the individual in the stages-of-change continuum. For example, for vaccine skeptics and deniers, the most suitable strategies seem to be those focused on emotional coping in the sense of including positive cues for vaccination in their preferred ways of facing reality. For individuals in the precontemplation and contemplation phases, a particular importance should be given to the ambivalent use of denial and to their real (and not imaginary) ability to ask for and use instrumental support. In the case of those in the stage of preparation, they could be stimulated into action via strategies supporting planning, positive reframing, and external influencers.

Furthermore, the transition from preclinical to clinical teaching in medical universities should not only be considered as a simple change of the study curriculum but as an opportunity for many students to commute from a more external-oriented thinking style to more autonomy and goal-oriented behavior. This in turn could be reflected in the attitude towards medical interventions and should be addressed in programs supporting HPV vaccination.

As a whole, these findings can be useful in designing more effective strategies, whether they be health policies or informal actions from influencers or other opinion leaders, in order to increase HPV vaccine acceptance.

## Figures and Tables

**Table 1:** Biographic variables.

Variables		Whole Sample (*N* = 1243)		Group 1 * (*N* = 196)		Group 2 ** (*N* = 721)		Group 3 *** (*N* = 326)	
A. Scale variables		Mean	SD	Mean	SD	Mean	SD	Mean	SD
Age		21.526	2.007	21.571	1.897	21.514	2.130	21.527	1.783
B. Nominal variables		*N*	%	*N*	%	*N*	%	*N*	%
Origin	urban	982	79.001	148	75.510	570	79.056	264	80.981
	rural	261	20.998	48	24.490	151	20.944	62	19.019
Year of study	1	174	13.998	35	17.857	101	14.008	38	11.656
	2	275	22.124	36	18.367	165	22.885	74	22.699
	3	268	21.561	35	17.857	162	22.469	71	21.780
	4	192	15.446	31	15.817	110	15.257	51	15.644
	5	164	13.194	23	11.735	91	12.621	50	15.337
	6	170	13.677	36	18.367	92	12.760	42	12.884
Country region	Bucharest	149	11.987	26	13.265	71	9.848	52	15.952
	South	276	22.205	42	21.428	163	22.607	71	21.779
	Northwest	679	54.625	103	52.552	407	56.449	169	51.840
	Northeast	139	11.183	25	12.755	80	11.096	34	10.429

* Group 1 = no intent of vaccination; ** Group 2 = precontemplation and contemplation; *** Group 3 = preparation. SD = standard deviation.

**Table 2:** HPV knowledge and psychological variables.

Variables		Whole Sample (*N* = 1243)		Group 1 * (*N* = 196)		Group 2 ** (*N* = 721)		Group 3 *** (*N* = 326)	
		Mean	SD	Mean	SD	Mean	SD	Mean	SD
Coping strategies	Self-distraction	5.576	1.476	5.637	1.480	5.535	1.471	5.628	1.486
	Active coping	6.759	1.152	6.719	1.202	6.718	1.156	6.874	1.109
	Denial	3.571	1.500	3.571	1.536	3.663	1.528	3.368	1.397
	Substance use	2.683	1.356	2.658	1.309	2.683	1.373	2.696	1.351
	Use of emotional support	5.353	1.696	5.505	1.672	5.341	1.654	5.288	1.798
	Use of instrumental support	5.844	1.615	5.811	1.614	5.876	1.606	5.794	1.639
	Behavioral disengagement	2.884	1.170	3.097	1.247	2.883	1.176	2.757	1.090
	Venting	5.298	1.646	5.530	1.702	5.227	1.615	5.316	1.672
	Positive reframing	5.846	1.472	5.586	1.552	5.834	1.473	6.027	1.400
	Planning	6.787	1.134	6.770	1.161	6.760	1.119	6.858	1.150
	Humor	5.546	1.856	5.352	1.821	5.533	1.869	5.690	1.843
	Acceptance	6.148	1.318	6.046	1.345	6.091	1.312	6.334	1.299
	Religion	4.546	1.968	4.903	2.126	4.474	1.966	4.490	1.853
	Self-blame	5.478	1.612	5.515	1.650	5.470	1.626	5.475	1.562
HPV knowledge		12.245	1.872	12.214	1.810	12.242	1.895	12.269	1.860
Health locus of control	Internal	25.823	4.723	25.663	4.868	25.771	4.778	26.036	4.516
	External (chance)	15.985	5.115	16.403	5.382	15.833	5.001	16.070	5.199
	External (powerful others)	23.385	5.274	21.928	5.452	23.299	5.281	24.450	4.922
Sense of coherence		49.134	9.739	48.270	8.937	49.233	9.799	49.435	10.064

* Group 1 = no intent of vaccination; ** Group 2 = precontemplation and contemplation; *** Group 3 = preparation. SD = standard deviation. HPV, Human Papilloma Virus.

**Table 3:** Statistical differences between the study groups *.

Variables		Sum of Squares	df	Mean Square	F	Statistical Significance	Post-Hoc Test	
							Mean Difference	*p*
Denial	Between groups	29.309	3	9.770	4.378	0.004	− 0.361	0.003 (group 2 − group 3)
	Within groups	2765.140	1239	2.232				
	Total	2794.449	1242					
Behavioral disengagement	Between groups	14.787	3	4.929	3.625	0.013	0.339	0.007 (group 1 − group 3)
	Within groups	1684.530	1239	1.360				
	Total	1699.318	1242					
Positive reinterpretation	Between groups	25.739	3	8.580	3.987	0.008	− 0.441	0.005 (group 1 − group 3)
	Within groups	2665.912	1239	2.152				
	Total	2691.651	1242					
Acceptance	Between groups	15.731	3	5.244	3.034	0.028	− 0.237	0.049 (group 2 − group 3)
	Within groups	2141.032	1239	1.728				
	Total	2156.763	1242					
Religion	Between groups	36.480	3	12.160	3.157	0.024	0.600	0.018 (group 1 − group 2)
	Within groups	4771.610	1239	3.851				
	Total	4808.090	1242					
Health locus of control (powerful others)	Between groups	796.737	3	265.579	9.750	0.001	− 1.523	0.026 (group 1 − group 2)
	Within groups	33747.676	1239	27.238			− 1.200	0.006 (group 2 − group 3)
	Total	34544.414	1242				− 2.522	0.001 (group 1 − group 3)

* only significant differences are displayed.

**Table 4:** Predictors of the vaccination intent

Effect		Model Fitting Criteria	Likelihood Ratio Tests		
		−2 Log Likelihood of Reduced Model	Chi-Square	df	Statistical Significance
Intercept		3054.108 ^a^	0.000	0	.
HPV knowledge		3054.528	0.420	3	0.936
Coping	Self-distraction	3055.586	1.479	3	0.687
	Active coping	3055.602	10.495	3	0.684
	Denial	3066.441	120.333	3	0.006
	Substance use	3059.637	50.529	3	0.137
	Use of emotional support	3057.981	30.873	3	0.275
	Use of instrumental support	3060.264	60.156	3	0.104
	Behavioral disengagement	3061.223	70.115	3	0.068
	Venting	3059.461	50.353	3	0.148
	Positive reframing	3065.513	110.405	3	0.010
	Planning	3057.736	30.628	3	0.305
	Humor	3054.381	0.273	3	0.965
	Acceptance	3056.343	20.235	3	0.525
	Religion	3064.931	100.823	3	0.013
	Self-blame	3056.918	20.810	3	0.422
Sense of coherence		3054.514	0.406	3	0.939
Health locus of control	Internal	3057.508	30.400	3	0.334
	Chance	3057.068	20.960	3	0.398
	Powerful others	3087.104	320.996	3	0.001
Year of study		3071.016	160.908	15	0.324

^a^ The reduced model was equivalent to the final model because omitting the effect did not increase the degrees of freedom.

**Table 5:** Preclinical–clinical differences ^a^.

Variables	2nd-Year Students		6th-Year Students		t	df	*p*
	Mean	SD	Mean	SD			
A. Group 1 *							
HPV knowledge	11.611	1.809	12.972	1.576	−3.403	70	0.001
B. Group 2 **							
HPV knowledge	11.957	1.842	12.891	1.607	−4.073	255	0.0005
C. Group 3 ***							
HPV knowledge	11.581	1.79	12.809	1.627	−3.669	114	0.0005
Active coping	6.878	1.02	7.285	0.918	−2.142	114	0.034
HLOC (powerful others)	24.972	4.385	22.761	4.471	2.591	114	0.011

* Group 1 = no intent of vaccination; ** Group 2 = precontemplation and contemplation; *** Group 3 = preparation. SD = standard deviation. HPV, Human Papilloma Virus, HLOC, health locus of control. ^a^ Only significant differences are figured.

## Data Availability

The dataset presented in this study is available on reasonable request from the corresponding author.
